# High-frequency multimodal training with a focus on Tai Chi in people with Parkinson’s disease: a pilot study

**DOI:** 10.3389/fnagi.2024.1335951

**Published:** 2024-02-15

**Authors:** Ketevan Toloraia, Ute Gschwandtner, Peter Fuhr

**Affiliations:** ^1^Department of Clinical Research and Neurology, University Hospital Basel, Basel, Switzerland; ^2^Department of Neurology, University Hospital Basel, Basel, Switzerland

**Keywords:** Parkinson’s disease, Tai Chi, cognitive decline, cognition, cognitive training, motor skills, neuropsychology, training

## Abstract

**Background and objectives:**

Cognitive decline is an important and common complication in patients with Parkinson’s disease (PD) since it significantly reduces the quality of life. A breakthrough in treating and preventing cognitive decline in PD remains to be achieved. This study aimed to evaluate the effectiveness of high-frequency and intensive multimodal training in improving motor and cognitive function.

**Methods:**

Twenty-eight patients diagnosed with idiopathic PD completed a comprehensive neuropsychological test battery and were neurologically examined. The patients of the intervention group (*n* = 15) underwent 2 weekly sessions of Tai Chi therapy over 4 weeks and participated in an individually tailored training program consisting of two modules (smartphone-based speech training and cognitive training). A matched control group consisted of *n* = 13 patients with PD who received computer-assisted cognitive training. The data were analyzed with repeated-measures ANOVA.

**Results:**

Four weeks of high-frequency training showed significant effects on verbal and figural episodic memory and visuospatial function in the intervention group . Compared to the control group, the cognitive performance of the intervention group improved significantly in visuospatial function and figural episodic memory. A significant improvement was also shown in the intervention group in the Tinetti Mobility Test and the Epworth Sleepiness Scale. The significant effects in the Tinetti mobility test remained after the 6 months follow-up. After the intervention, the patients reported high motivation and satisfaction with the multimodal training.

**Conclusion:**

In patients with PD, a multimodal training program not only improves gait and stability but may also contribute to improving cognition.

**Clinical trial registration:**

ClinicalTrials.gov Identifier: NCT04103255; https://register.clinicaltrials.gov/prs/app/action/LoginUser?ts=1&cx=-jg9qo4.

## Introduction

Parkinson’s disease (PD) is a progressive neurodegenerative disorder (Poewe et al., 2017) with a multisystem nature and a broad spectrum of symptoms and signs (Titova et al., 2017). Its complex underlying pathology includes degeneration of dopaminergic and non-dopaminergic pathways leading to motor and a wide range of non-motor symptoms (NMS) (Titova et al., 2017). Bradykinesia, resting tremor, rigidity, and instability define the core syndrome (Ferrazzoli et al., 2018; Barboza et al., 2019; Liu et al., 2019), while cognitive decline, anxiety, apathy, depression, psychosis, and impulse control disorders are the common NMS (Aarsland et al., 2021; Weintraub et al., 2022). The NMS may negatively affect a patient’s quality of life (QoL) even more than motor symptoms (Khoo et al., 2013).

In recent decades, interest in the QoL of people with PD has increased, with the clinical focus shifting from how well patients move to how well they live (Chen et al., 2020). Dopaminergic medications are helpful throughout the course of this illness to treat motor symptoms (Ferrazzoli et al., 2018); however, they can induce side effects (Bernal-Pacheco et al., 2012). The NMS rarely responds to dopaminergic therapy, and only limited evidence supports pharmacological treatment and invasive interventions. However, non-pharmacological interventions to improve both motor symptoms and NMS offer an increasingly accepted alternative (Feng et al., 2020).

Cognitive decline is a significant and frequent complication of PD; approximately 27% of non-demented PD patients have a mild neurocognitive disorder (Mild Cognitive Impairment, MCI) (Chaudhuri et al., 2011; Litvan et al., 2012; Walton et al., 2017), and up to 80% of these patients proceed to develop PD dementia (PD-D) in the long term (Reichmann et al., 2009), especially when combined with olfactory dysfunction (Heinzel et al., 2016; Cozac et al., 2017). The cognitive course in PD can be heterogeneous and may affect visuospatial function, attention, executive function, memory, and language (Litvan et al., 2012). Attention-related cognitive impairment (dual-tasking dysfunction) affects motor skills and daily activities and is associated with an increased risk of falls (Emre et al., 2004; Price and Shin, 2009; Petrelli et al., 2014; Zimmermann et al., 2014). Besides anti-dementia drugs such as rivastigmine (Emre et al., 2004) non-pharmacological approaches have proven valuable (Zimmermann et al., 2014; Walton et al., 2017; Vergara-Diaz et al., 2018).

Motor dysfunctions exhibited by PD patients, such as start hesitation and freezing of gait, are included in the cardinal motor symptoms with bradykinesia, tremor, rigidity, and postural instability (Díez-Cirarda et al., 2018). The early loss of dopamine in PD leads to reduced automatic control of movements and increased involvement of the frontal lobe circuits to exude cognitive control. As a result, individuals with PD must manage and maintain a greater cognitive load to perform motor tasks (Vergara-Diaz et al., 2018).

A negative relationship between risk for PD and physical activity was first supposed by Sasco et al. (1992). Several research studies subsequently confirmed a positive effect of physical activity on motor and non-motor symptoms of PD (Amara and Memon, 2018). Tai Chi is a body–mind exercise which originated in traditional Chinese medicine. By virtue of being a form of meditative physical activity containing a high cognitive component, Tai Chi has the potential to treat both motor and NMS in PD equally and positively impact the quality of life (Nocera et al., 2010; Kurt et al., 2018; Liu et al., 2019; Chen et al., 2020; Deuel and Seeberger, 2020; Khuzema et al., 2020).

PD is recognized as a complex disease process. Therefore, multidisciplinary rehabilitation that addresses PD patients’ motor and non-motor aspects is encouraged (Lo Buono et al., 2021). Numerous studies exist regarding NMS, such as cognitive training or motor symptoms, particularly gait or balance; however, fewer studies combine both motor and non-motor training and take an individualized approach to treating and preventing PD. Furthermore, there are no studies combining non-motor and motor training, such as Tai Chi, with speech training for the treatment of PD. However, our study provides a novelty to the research by including the components of Tai Chi and speech training.

Due to the complex clinical picture of PD, a combined, multimodal study is highly recommended (Titova et al., 2017) since research has shown that multimodal training improves cognitive function more than a purely cognitive training program (Reuter et al., 2012).

This study aims to compare the effects of high-frequency and intensive multimodal training, consisting of Tai Chi, computer-assisted cognitive training (CogniFit) (Shatil, 2013), and computer-assisted speech training (SpeechCare)(Starke and Mühlhaus, 2018) with modal cognitive training alone, including computer-assisted cognitive training (CogniPlus) (Zimmermann et al., 2014).

The following questions were investigated: Are cognitive functions improved by a multimodal program? Are the effects of a multimodal treatment, a cognitive treatment in combination with a motor and speech treatment, significantly better than those of a purely cognitive treatment? We hypothesize that an individualized high-frequency and intensive multimodal program can improve cognitive function in patients with PD, and the improvements can be maintained after a follow-up period of 6 months. We also hypothesize that the individualized multimodal training program will be more effective in improving cognitive function than only modal cognitive training.

## Methods

### Participants

Between July 2020 and July 2021, 21 patients were recruited into the intervention group from the Movement Disorders Clinic of the University Hospital Basel and patients via advertisements and referrals from other clinics. Patients were included if they met the UK Parkinson’s Disease Brain Bank criteria (Gibb and Lees, 1988). Patients were excluded if they had PD with onset at a young age (less than 18 years), had moderate or severe dementia, severe neurological conditions additional to PD, or were not proficient in German. Deep brain stimulation (DBS) was not used as an exclusion criterion. Two patients were excluded for personal and health reasons from the first follow-up. One patient was excluded from the second follow-up for health reasons. Fifteen patients were included in the intervention group sample for the final analysis.

**Figure 1:**
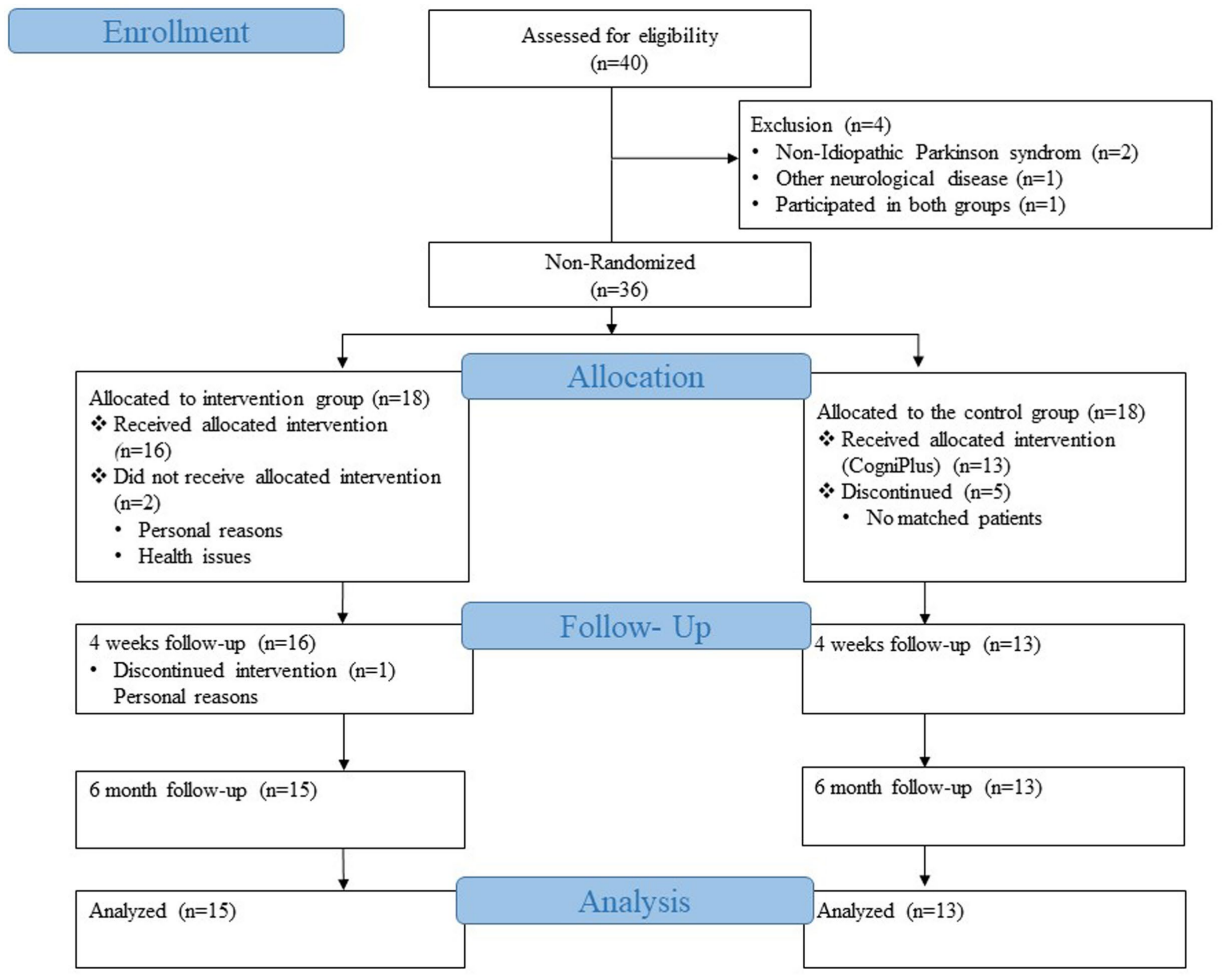
CONSORT flow diagram modified for non-randomised trial design.

Our control group consisted of patients from an already completed and published study of the Movement Disorders Clinic of the University Hospital Basel (Zimmermann et al., 2014). Patients in the control group were recruited from May 2011 to January 2013 from the outpatient clinic for movement disorders of the University Hospital Basel or via advertisements in the journal of the Swiss Parkinson’s Association. A total of 39 patients were recruited, who were then randomly divided into two groups. Twenty patients were trained with Nintendo Wii programs, the game console with motion-sensitive controllers, and the other 19 patients received the cognitive training program CogniPlus. The group that received the CogniPlus cognitive training program was then chosen as the control group for the current study since the aim was to compare two conditions: on the one hand, the multimodal training that included the cognitive training, and on the other hand, the purely cognitive training. The inclusion and exclusion criteria for the control group were the same as for the intervention group (Zimmermann et al., 2014). One of the 19 patients from the CogniPlus group was excluded because he participated in the intervention group of our current study, and study participants are not allowed to be part of both the intervention and control group. Therefore, the patient was excluded from the control group. Of the 18 patients, 13 were selected for the control group after careful matching with the intervention group (Figure 1).

In all participants, the dose of levodopa remained unchanged during the intervention.

### Study design

**Figure 2:**
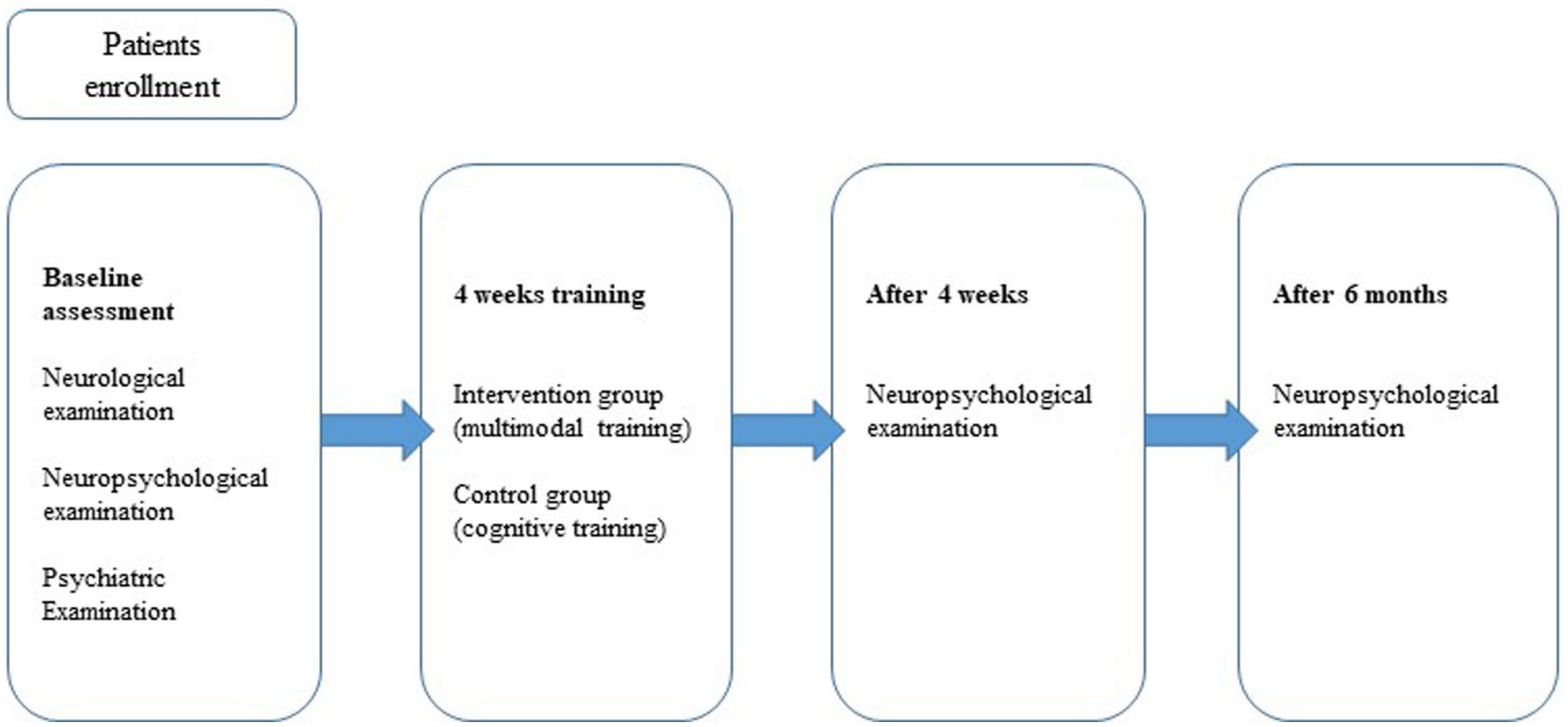
Study design.

The study design was a non-randomized controlled trial with a follow-up period of 6 months (Figure 2). The intervention and control groups were matched for age, gender, education level, disease duration, levodopa equivalent daily dose (LEDD) (Tomlinson et al., 2010), motor signs (UPDRS III) (Fahn et al., 1987), Montreal Cognitive Assessment (MoCA) (Nasreddine et al., 2005), Beck Depression Inventory (BDI-II) (Hautzinger et al., 2006), and Beck Anxiety Inventory (BAI) (Beck et al., 1988; Table 1).

**Table 1:** Participant’s characteristics at baseline.

	**HIPP median (Q.25 – Q.75)**	**CogniPlus median (Q.25 – Q.75)**	**Mann–Whitney Test** *p-value*
No. of patients	15	13	
Age, y	63 [59, 75]	68 [63, 73]	0.62
Sex (female: male)	4:11	3:10	0.59
Education, y	18 [13, 18]	16.00 [13, 17]	0.18
Disease duration	12 [8, 16]	11.0 [6, 14]	0.25
LEDD	399 [100, 575]	550 [283, 806]	0.20
UPDRS III	13 [5, 21]	15 [4, 21]	0.89
MoCA	25 [24, 29]	27 [25, 28]	0.25
BDI-II	9 [5, 12]	6 [2, 10]	0.11
BAI	7 [4, 15]	7 [5, 9]	0.72

All values represent median [quartiles]. Quartiles refer to the 25th and the 75th percentile. HIPP, high frequency and intensive prevention program; CogniPlus, computer-assisted cognitive training; LEDD, Levodopa-equivalent doses; UPDRS III, Unified Parkinson’s Disease Rating Scale, Motor Subscale; MoCA, Montreal Cognitive Assessment; BDI-II, Beck Depression Inventory-II; BAI, Beck Anxiety Inventory.

Eighteen patients from the intervention group completed a comprehensive neuropsychological and neuropsychiatric test battery at the study’s baseline and underwent a neurological examination. Two patients dropped out at follow-up 1 due to health and personal reasons, and one patient was unable to participate after the 6-month follow-up due to health reasons. In the end, 15 patients could be reconsidered for the analyses.

Initially, the data of 19 patients from the already completed study were available for the control group. After applying the exclusion criteria and the matching process with the intervention group, 13 patients remained for the analysis. CogniPlus for the control group included 3 sessions per week for 4 weeks.

Patients in the intervention and control groups were assessed for cognitive function after the intervention (FU 1) and 6 months after the intervention (FU 2) using a comprehensive neuropsychological test battery.

### The intervention group

Patients in the intervention group participated in a high-frequency and intensive multimodal program for 4 weeks. In this context, high frequency means that the training took place several times a week, and intensive means that three different interventions (Tai Chi, cognitive training, and speech training) were used in combination to form an intensive training.

Except for Tai Chi, which was conducted twice a week, the frequency and duration of training modules were modular, their level being adapted individually according to the initially measured severity of cognitive and language deficits. The high-frequency and intensive prevention program included the following training sessions:

1. Tai Chi – Keep Moving® is an exercise program for patients with movement disorders that explicitly utilizes the health aspect of Tai Chi movement teachings. Each session lasted 60 min and ended with a 10-min meditation. The Tai Chi program included: (1) Gentle gymnastics and stretching exercises; (2) Standing exercises for balance and posture; (3) Gait training for control and balance; (4) Range of motion exercises for motor skills and coordination; and (5) Meditation for a positive mood. The details of this program can be found on the following website: https://taiji-therapie.ch/. The Tai Chi sessions in our study were conducted twice a week under the guidance of certified Tai Chi instructors. The trainers gave individual feedback to each participant. Each participant also received a DVD with the Tai Chi units and could practice at home in case they preferred.
2. CogniFit® – is an online-based personalized cognition training program. This well-validated cognitive training improves attention, working memory, memory, executive function, and visual perceptual functions. Each session lasted about 15 min and was applied 2–5 times per week based on the results of the BL tests. The home-based approach took place under the supervision of a psychologist or a specially trained psychology student. Performance feedback and cumulative scores were displayed so that users could track their progress. The program itself automatically sets the level of difficulty. The study leader got the information from the participants’ progress reports. Details of the program can be found on the following website: https://www.cognifit.com/.
3. SpeechCare – (MoveApp) is a home-based approach for patients with PD. The training program uses automatic speech recognition (ASR) and thus can provide feedback on speech intelligibility. Each session lasted 12 min and was conducted 1–3 times per week based on the results of the BL test of SpeechCare (Starke and Mühlhaus, 2018). The domestic approach took place under the supervision of a psychologist or a specially trained psychology student. The level of difficulty was manually adjusted as needed. Details of the program can be found on the following website: https://www.speechcare.de/.

The intervention group participated in Tai Chi sessions. Part of the Tai Chi sessions were conducted live at the University Hospital Basel, and the other part was conducted online via video call due to the COVID-19 pandemic restrictions.

At the beginning of the intervention, the patients received iPads with instructions on using them and individual schedules. The iPad ran the speech and cognitive training programs. The patients could also participate in the Tai Chi sessions using iPads. Those who could not use the iPads received help from psychology students.

After the four-week intervention, patients completed a questionnaire to assess their overall satisfaction with the multimodal approach and the individual intervention programs. After the intervention, the patients were tested (Follow-up 1), and after 6 months, they were retested (Follow-up 2).

Standard protocol approvals, registrations, and patient consent were implemented. The study was registered on ClinicalTrial.gov (NCT04103255) and approved by the local ethics committee (Ethikkommission beider Basel, 2019–01834). All participants were informed of the nature of the study and gave written consent to participate.

### The control group

The patients in the control group received a computer-assisted cognitive training program for 4 weeks: CogniPlus (Schuhfried GmbH, Vienna, Austria). CogniPlus is research-based and has an up-to-date psychological assessment. Each training session consisted of different tasks (Attention, Memory, Executive function, and Visuomotor function) that were performed for 10 min each. Like the cognitive training in the intervention group, in the control group, the difficulty level was adapted automatically by the program itself.

The patients in the control group were also tested again after the cognitive training (follow-up 1), and another (Follow-up 2) test followed after 6 months. Details of the program can be found on the following website: https://www.schuhfried.com/cogniplus/. The training sessions were supervised by a psychologist or a specially trained psychology student.

## A neuropsychological examination

The comprehensive neuropsychological examination included the following cognitive domains: attention, working memory, executive functions, verbal fluency, episodic memory, and visuospatial functions: **Attention:** Trail Making Test, part A; Stroop Color-Word Test: time for color naming; Executive Functions: Stroop Color-Word Test: time for interference task divided by the time for color naming; Trail-Making Test: time for part B divided by the time for part A; Wisconsin Card Sorting Test: correct categories/number of errors. Fluency: Semantic verbal fluency test: correct answers; Phonemic verbal fluency: correct answers; Five-Point test: correct answers. Verbal and Figural Episodic **Memory:** Rey-Osterrieth Complex Figure (ROCF): savings (immediate recall; delayed recall); California Verbal Learning Test (German version): short-delayed recall, long-delayed recall. **Working Memory:** Corsi blocks from the German version of the Revised Wechsler Memory Scale: correct forwards/correct backward. Digit span from the German version of the Revised Wechsler Memory Scale: correct forwards/correct backward. Visuospatial Functions: Mosaic test; Rey-Osterrieth Complex Figure Copy.

The raw scores of the tests were normalized and transformed into adjusted z-scores (Berres et al., 2000). All neuropsychological tests were performed during the patients’ ON phase (under dopamine treatment).

### Neurological assessment

The motor involvement was assessed with Unified Parkinson’s Disease Rating Scale (Movement Disorder Society Task Force on Rating Scales for Parkinson’s Disease, 2003).

## Statistical procedure

Age, education level, disease duration, LEDD and UPDRS III, MoCA, BDI, BAI, and neuropsychological performance of the two groups at BL were compared using the Mann–Whitney *U* and Chi-square tests for the sex variable.

To examine changes over time, a mixed-model repeated-measures analysis of variance (ANOVA) was performed. A group-time interaction was used to compare the intervention and control groups. Bonferroni correction was used for multiple corrections. The Wilcoxon signed rank test and Friedman test was performed to test the effects of the intervention on motor function and daytime sleepiness variables, both of which had a non-normal distribution.

Paired t-tests were used to examine within-group changes between baseline to 4 weeks. A significance threshold of 5% (*p* = 0.05) with a 95% confidence interval (CI) was applied for these analyses.

Statistical analyses were performed using IBM SPSS Statistics, version 28.0.1.0.

## Data availability

The datasets generated and analyzed during the current study are not publicly available but are available from the corresponding author.

### Results

### Confounding factors and neurological parameters at BL

The demographic data of the sample, including MoCA scores, age, sex, medication, UPDRS III, age at disease onset, duration of disease, depression, and anxiety are given in Table 1. There was no statistically significant variation between these two groups on all these variables.

### Comparison of training effects within groups

A comparison of training effects on neuropsychological variables, including 95% confidence intervals (CIs), is shown in Table 2. In the intervention group, change scores (BL to FU1) revealed significant differences in verbal episodic memory:


$$\begin{array}{l} BaselverballearningTest-shortdelayedrecall:\\ \eta p^{2}=0.34;p=0.02\;\left[95\%\mathrm{CI}-1.90;-0.22\right]; \end{array}$$

$$\begin{array}{l} BaselverballearningTest-longdelayedrecall:\\ \eta p^{2}=0.47;p=0.00\;\left[95\%\mathrm{CI}-0.91;-0.23\right], \end{array}$$

figural episodic memory: Rey Osterrieth Complex Figure Test ( ROCF) , immediate and delayed recall:


$$\eta p^{2}=0.46;p=0.00\;\left[95\%\mathrm{CI}-0.83;-0.19\right];$$

$$\eta p^{2}=0.61;p=<0.00\;\left[95\%\mathrm{CI}-1.09;-0.41\right]\;$$

and visuospatial function:


$$ROCFcopy:\;\;\eta p^{2}=0.25;p=0.05\;\left[95\%\mathrm{CI}-1.44;-0.00\right];$$

$$MosaicTest:\eta p^{2}=0.25;p=0.049\;\left[95\%\mathrm{CI}-2.44;-0.01\right].$$

**Table 2:** Cognitive tests at baseline and after 4 weeks of intervention.

	**Group**	**T0 M (SD)**	**T1 M (SD)**	**T0-T1 M (SD)**	**P (95% CI)**	**Time effect (Effect size)**	**Time × Treatment (Effect size)**
**Attention**							
TMT (A)	HIPP	−0.35 (1.52)	−0.28 (1.25)	−0.07 (0.99)	0.79 (−0.62; 0.48)	*p* = 0.790 (0.003)	*p* = 0.893 (0.001)
	CogniPlus	−0.41 (0.74)	−0.39 (0.97)	−0.02 (0.73)	0.91 (−0.46; 0.42)		
Stroop Test	HIPP	−0.63 (1.88)	−1.02 (1.44)	0.39 (1.33)	0.28 (−0.35; 1.13)	*p* = 0.372 (0.031)	*p* = 0.340 (0.035)
	CogniPlus	−0.58 (1.44)	−0.57 (0.90)	−0.01 (0.71)	0.95 (−0.44; 0.42)		
**Executive function**							
Stroop Test	HIPP	0.63 (1.08)	0.57 (0.84)	0.05 (1.22)	0.86 (−0.62; 0.73)	*p* = 0.815 (0.002)	*p* = 0.980 (0.000)
	CogniPlus	0.35 (1.03)	0.31 (0.90)	0.04 (0.97)	0.87 (−0.54; 0.63)		
TMT (B/A)	HIPP	−1.43 (3.17)	−1.9 (3.76)	0.47 (3.51)	0.61 (−1.47; 2.41)	*p* = 0.691 (0.006)	*p* = 0.612 (0.010)
	CogniPlus	−0.43 (1.24)	−0.37 (1.01)	−0.06 (1.17)	0.86 (−0.77; 0.65)		
mWCST	HIPP	−0.56 (1.56)	−0.28 (0.76)	−0.27 (1.62)	0.52 (−1.17; 0.62)	*p* = 0.441 (0.023)	*p* = 0.678 (0.007)
	CogniPlus	−0.55 (1.14)	−0.46 (1.13)	−0.08 (0.28)	0.31 (−0.2.5; 0.08)		
**Verbal fluency**							
Semantic verbal fluency	HIPP	−1.20 (1.13)	−0.85 (1.01)	−0.35 (1.06)	0.22 (−0.94; 0.24)	*p* = 0.027 (0.174)	*p* = 0.661 (0.007)
	CogniPlus	−0.81 (1.17)	−0.3 (0.72)	−0.51 (0.86)	0.053 (−1.03; 0.01)		
Phonemic verbal fluency	HIPP	0.05 (0.74)	−0.17 (0.72)	0.22 (0.55)	0.13 (−0.08; 0.53)	*p* = 0.781 (0.003)	*p* = 0.155 (0.076)
	CogniPlus	−0.33 (1.02)	0.00 (1.18)	−0.34 (1.34)	0.39 (−1.17; 0.49)		
Five-Point test Correct answers	HIPP	−0.56 (1.03)	−0.56 (0.97)	0.00 (1.03)	0.99 (−0.56; 0.58)	*p* = 0.559 (0.011)	*p* = 0.584 (0.012)
	CogniPlus	−0.68 (1.14)	−0.48 (1.44)	−0.19 (0.84)	0.42 (−0.70; 0.31)		
**Figural episodic and verbal memory**							
ROCF (Immediate recall)	HIPP	−0.15 (1.42)	0.36 (1.47)	−0.51 (0.57)	0.00 (−0.83; −0.19)	*p* = 0.003 (0.284)	*p* = 0.240 (0.053)
	CogniPlus	−0.24 (0.85)	−0.01 (1.35)	−0.23 (0.66)	0.23 (−0.63; 0.17)		
ROCF (Delayed recall)	HIPP	−0.16 (1.39)	0.59 (1.15)	−0.76 (0.446)	<0.00 (−1.09; −0.41)	p = <0.001 (0.396)	*p* = 0.045 (0.146)
	CogniPlus	−0.29 (0.85)	−0.04 (1.35)	−0.24 (0.066)	0.21 (−0.65; 0.16)		
BVLT (Short delayed recall)	HIPP	−0.45 (1.81)	0.61 (0.98)	−1.06 (1.56)	0.02 (−1.90; −0.22)	*p* = 0.009 (0.244)	*p* = 0.271 (0.048)
	CogniPlus	−0.90 (1.44)	−0.43 (0.85)	−0.46 (1.18)	0.20 (−1.21; 0.29)		
BVLT (Long delayed recall)	HIPP	0.01 (0.94)	−57 (0.77)	−0.56 (0.61)	0.00 (−0.91; −0.23)	p = <0.001 (0.451)	*p* = 0.450 (0.023)
	CogniPlus	−0.87 (0.80)	−0.07 (1.00)	−0.79 (0.94)	0.01 (−1.39; −0.20)		
**Working memory**							
Corsi blocks (correct forwards)	HIPP	0.14 (1.36)	0.05 (1.3)	0.09 (1.50)	0.82 (−0.74; 0.92)	*p* = 0.690 (0.006)	*p* = 0.436 (0.023)
	CogniPlus	−0.45 (1.08)	−0.15 (1.24)	−0.50 (1.17)	0.15 (−1.21; 0.20)		
Corsi blocks (correct backwards)	HIPP	−0.04 (1.9)	1.07 (1.25)	−0.15 (1.09)	0.61 (−0.75; 0.46)	*p* = 0.340 (0.035)	*p* = 0.842 (0.002)
	CogniPlus	0.05 (0.97)	0.27 (1.15)	−0.22 (0.911)	0.39 (−0.77; 0.32)		
DSST (correct forwards)	HIPP	0.09 (1.15)	0.13 (1.06)	−0.04 (1.21)	0.90 (−0.70; 0.62)	*p* = 0.482 (0.019)	*p* = 0.371 (0.031)
	CogniPlus	0.27 (0.91)	−0.04 (0.94)	0.31 (0.78)	0.17 (−0.15; 0.78)		
DSST (correct backwards)	HIPP	0.09 (1.15)	0.13 (1.06)	0.12 (0.78)	0.56 (−0.31; 55)	*p* = 0.794 (0.003)	*p* = 0.571 (0.012)
	CogniPlus	0.17 (0.83)	−0.12 (1.06)	−0.04 (0.72)	0.83 (−0.48; 0.39)		
**Visuospatial functions**							
Mosaic Design Test	HIPP	0.18 (1.46)	1.4 (2.62)	−1.22 (2.19)	0.049 (−2.44; −0.01)	*p* = 0.079 (0.114)	*p* = 0.048 (0.142)
	CogniPlus	0.13 (1.08)	0.05 (1.23)	0.08 (0.56)	0.63 (−0.26; 0.42)		
ROCF (Copy)	HIPP	−0.87 (1.25)	−0.15 (1.30)	−0.72 (1.29)	0.050 (−1.44; −0.00)	*p* = 0.113 (0.094)	*p* = 0.157 (0.076)
	CogniPlus	−0.73 (1.31)	−0.68 (1.46)	−0.04 (1.14)	0.90 (−0.73; 0.64)		

CI, confidence interval; Q = quartile; T0 = Baseline; T1 = 4-week follow-up; TMT = Trail Making Test; mWCST = modified Wisconsin Card Sorting Test; ROCF = Rey–Osterrieth Complex Figure Test; BVLT = Basel Verbal Learning Test; DSST = Digit Symbol Substitution Test. The *p-*value was adjusted for multiple testing: Bonferroni; ^*^p < 0.05.

In the control group, change scores revealed significant differences in *Basel verbal learning Test − long delayed recall*:


$$\eta p^{2}=0.44;p=0.01\;\left[95\%\mathrm{CI}-1.39;-0.20\right].$$

**Figure 3:**
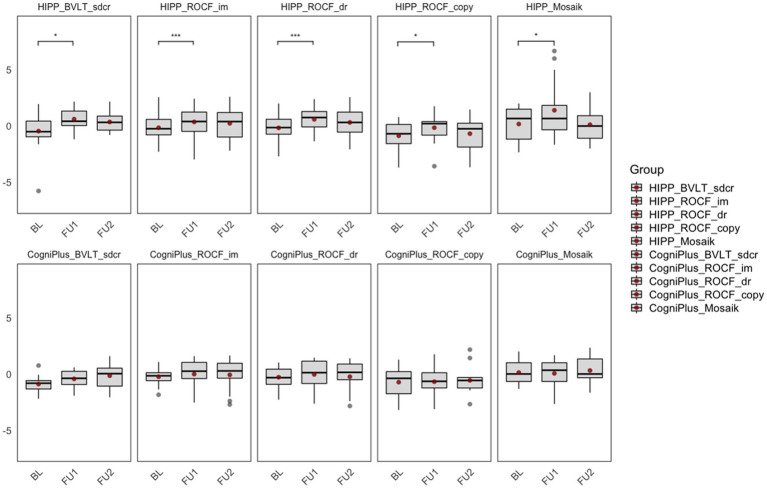
Comparison of training effects within groups. BL, Baseline; FU1, Immediately after the intervention; FU2, 6-month follow-up after the intervention; HIPP_BVLT_sdcr, intervention group, Basel Verbal Learning Test, short delayed recall; HIPP_ROCF_im, intervention group, Rey–Osterrieth Complex Figure Test, immediate recall; HIPP_ROCF_dr, intervention group, Rey–Osterrieth Complex Figure Test, delayed recall; HIPP_ROCF_copy, intervention group, Rey–Osterrieth Complex Figure Test, Copy; HIPP_Mosaik, intervention group, Mosaic Design Test; CogniPlus_BVLT_sdcr, control group, Basel Verbal Learning Test, short delayed recall; CogniPlus_ROCF_im, control group, Rey–Osterrieth Complex Figure Test, immediate recall; CogniPlus_ROCF_dr, control group, Rey–Osterrieth Complex Figure Test, delayed recall; CogniPlus_ROCF_copy, control group, Rey–Osterrieth Complex Figure Test, Copy; CogniPlus_Mosaik, control group, Mosaic Design Test. *p ≤ 0.05; **p < 0.01; ***p < 0.001.

The results are shown in Figure 3.

### Comparison of training effects between groups

A comparison of training effects in the neuropsychological variables is shown in Table 2. Comparison of the variables in the time x treatment condition (between groups) revealed significant differences in visuospatial function: Mosaic Design test: $\eta p^{2}=0.14,p=0.048;$ and in figural episodic memory: Rey Osterrieth Complex Figure Test, delayed recall: $\eta p^{2}=0.15,p=0.045$ .

In the *Basel verbal learning Test − long delayed recall*, both groups showed a significant improvement in change scores (BL to FU1). However, the significant difference between the two groups already existed at baseline.

In the domains of attention, executive function, verbal fluency and working memory, we found no statistically significant differences between or within groups.

### Additional tests in the intervention group

**Figure 4:**
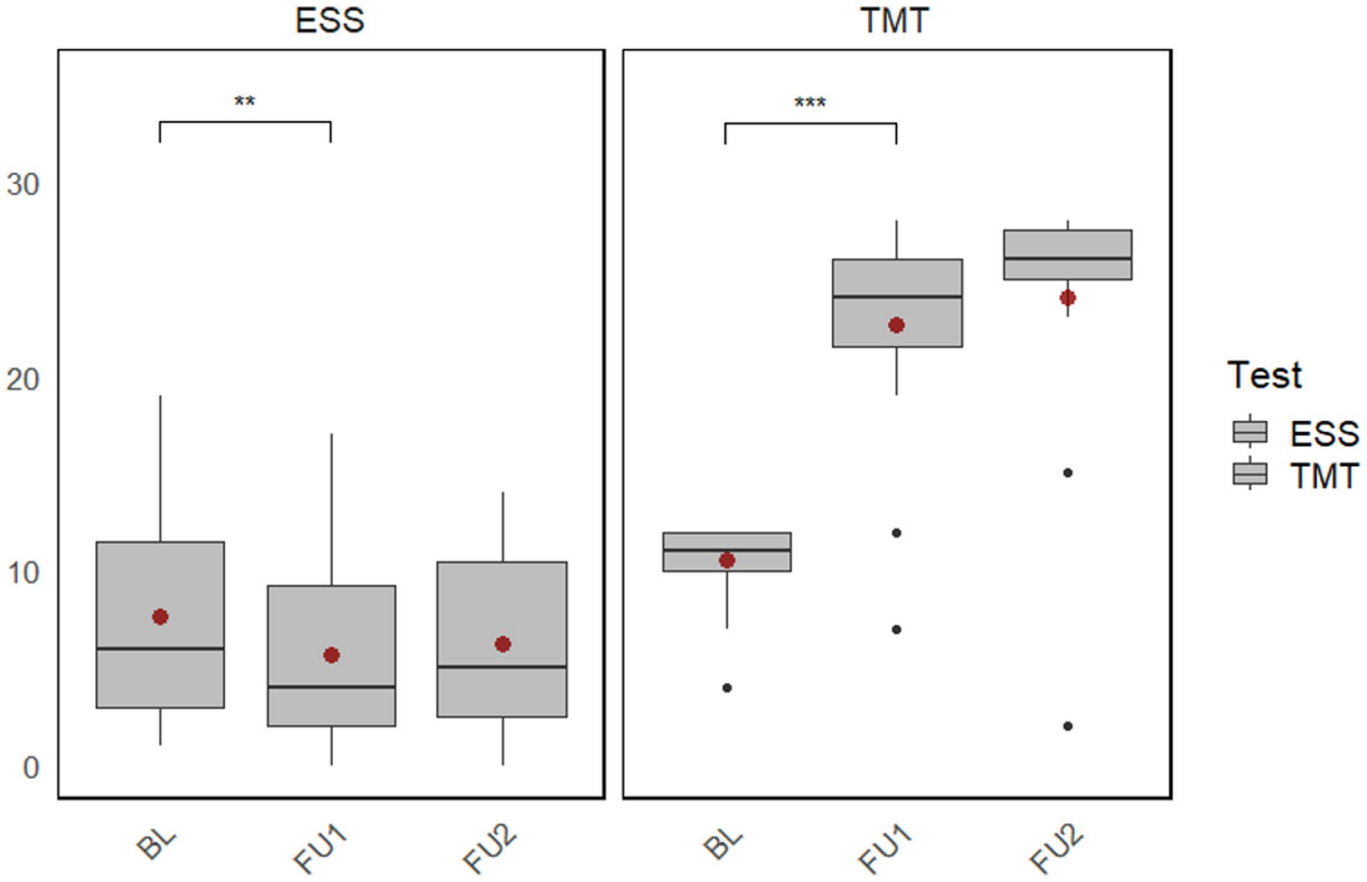
Plot of the changes within the intervention group (baseline to follow-up 1 and follow-up 2) in the Tinetti Mobility Test and Epworth Sleepiness Scale. BL, Baseline; FU1, Immediately after the intervention; FU2, 6-month follow-up after the intervention; ESS, Epworth Sleepiness Scale; TMT, Tinetti Mobility Test. **p < 0.01; ***p < 0.001.

The intervention group was additionally examined with regard to motor functions and daytime sleepiness, which was regrettably not possible in the control group, which originates from our former study. The results show that the Tinetti Mobility Test, a valid test for assessing falls and balance in PD patients, improved significantly after the multimodal training program ( r=0.60;p<0.001 ). The improvement persisted after 6 months. The improvement was also shown in the Epworth Sleepiness Scale so that the patients had significantly less daytime sleepiness after the multimodal training ( r=0.53;p=0.01 ) (Figure 4).

## Discussion

The aim of this study was to determine whether a high-frequency and intensive multimodal training program, including Tai Chi as an element of multimodal training, is suitable for improving cognitive function in Parkinson’s disease patients and whether it is significantly different from cognitive training in a control group.

There are numerous studies regarding NMS, such as cognitive training, speech training, or motor symptoms, in particular, gait or balance; however, there are fewer studies that combine both motor and non-motor training and take an individualized approach, which is highly recommended due to the complex clinical picture of PD (Titova et al., 2017).

The current study shows that a multimodal training program can improve cognitive performance in PD patients and that it can also help improve gait and balance and reduce daytime sleepiness.

Reuter et al. (2012) showed that multimodal cognitive training with physical activity seems more effective than cognitive training programs without motor training in patients with PD. In addition, they suggested that varied and challenging training is more effective than only cognitive training.

Oswald et al. (1996) showed that combined psychomotor and cognitive training led to an improvement in psychomotor performance and cognitive performance. In contrast, psychomotor training and memory training alone did not lead to such effects.

The intervention group showed a significant improvement in verbal and figural episodic memory.

Verbal memory deficits in PD patients are related to executive dysfunction (especially encoding), attention, and working memory deficits (Brønnick et al., 2011; Chiaravalloti et al., 2014). Deficits in episodic memory and visuospatial functions indicate early cognitive changes in patients with PD and increase the risk for early progression to dementia (Aarsland et al., 2021). Episodic memory impairment reduces functioning, is associated with neuropsychiatric symptoms, and decreases the quality of life (Pourzinal et al., 2021).

Chung et al. (2021) showed that episodic memory was associated with motor skills. Overall improvement in motor skills was associated with improved episodic memory in PD. Multimodal training enables the improvement of motor skills along with episodic memory in the intervention group.

The intervention group also showed significantly improved visuospatial functions compared to the control group. Lam et al. (2011) proved that Tai Chi training significantly improved visual attention in older adults at risk of progressive cognitive decline.

In addition, a significant reduction in daytime sleepiness was observed in the HIPP group. Yang and colleagues showed the same result (Yang et al., 2017). They observed that Tai Chi groups (individual Tai Chi or group-based Tai Chi therapy) improved global non-motor symptoms (NMS) and sleep compared to BL. Qigong, which involves meditative movement such as Tai Chi, has also shown significant benefits for nighttime sleep quality in people with PD. In this outcome, meditation may play an important role (Moon et al., 2017).

In the intervention group, beneficial effects of Tai Chi on motor deficits were observed. Those findings are further validated by research by Li et al. (2012), who showed that Tai Chi leads to better outcomes than physical activity, resistance training, or stretching. Additionally, Tai Chi has previously been shown to improve gait and balance and to reduce the fall risk in people with PD (Liu et al., 2019).

Patients with normal cognition or mild cognitive impairment benefit more from cognitive training than patients with more advanced cognitive impairment (Reuter et al., 2012). Also, depression and anxiety can influence performance in neuropsychological tests. In our study, there were patients without dementia, and there were no significant differences between the groups regarding depression or anxiety.

### Study limitations and strengths

The study has some limitations that must be addressed. A limitation of the present study is the relatively small sample size. Furthermore, the study is limited by the fact that the intervention group received more training sessions per week than the control group. The frequency of training sessions could influence the result (Caciula et al., 2016), however, the high intensity of training in itself may be an important factor contributing to success. In addition, the intervention and control groups were carefully matched rather than randomized, as the two groups were not evaluated in the same period, potentially biasing the sample. Another aspect to consider is that this study only examined multimodal training as a whole and not the individual components (e.g., Tai Chi, cognitive training and speech training) separately. As a result, it is difficult to determine which components of multimodal training are effective on their own and which have the strongest effect. This should be investigated in further research. In addition, a more detailed analysis of treatment effects could provide a clearer understanding of the caudate and putamen circuit and interventions in people with PD.

The study also has several strengths. First, this study implemented a multimodal training approach, combining motor and non-motor training with a focus on Tai Chi. This combination of interventions, which is new to our knowledge, is a novelty in research and shows considerable potential for improving motor and cognitive function in PD patients. Second, two follow-up assessments were conducted to investigate the continued effectiveness of the intervention program immediately after the intervention and after 6 months. Third, the low number of dropouts indicates high adherence to the intensive training program, suggesting that it is feasible and can be implemented in non-demented PD patients with reasonable effort. Finally, the high-frequency and intensive multimodal program can be used as a home treatment method which is quite helpful, as PD patients usually have a higher acceptance rate of home treatment (Yang et al., 2017). Furthermore, this study developed and used a questionnaire to assess the participants’ satisfaction with the programme, which showed a high level of satisfaction with the multimodal training and Tai Chi in particular.

### Directions for future research

Future research should further investigate the effectiveness of high-frequency and intensive multimodal training in preventing PD progression and stabilizing or even reducing symptoms, thereby improving the quality of life of PD patients. In addition, studies with multiple follow-up assessments are required to explore how long the beneficial effects of high-frequency and intensive multimodal training in PD last.

## Conclusion

This investigation supports future studies into the efficacy of multimodal training, including Tai Chi, to improve motor and cognitive abilities in patients with PD.

## Data availability statement

The data that support the findings of this study are available from the corresponding author, upon reasonable request.

## Ethics statement

The studies involving humans were approved by Ethics committee of North/West Switzerland. The studies were conducted in accordance with the local legislation and institutional requirements. The participants provided their written informed consent to participate in this study.

## Author contributions

KT: Data curation, Formal analysis, Investigation, Methodology, Project administration, Validation, Writing – original draft. UG: Conceptualization, Formal analysis, Funding acquisition, Methodology, Supervision, Writing – review & editing. PF: Conceptualization, Formal analysis, Funding acquisition, Methodology, Supervision, Writing – review & editing, Resources, Validation.
